# The current state of refractive surgery

**Published:** 2017

**Authors:** Calin Petru Tataru

**Affiliations:** * National Editorial Advisory Board, Bucharest, Romania

The modern refractive surgery is based on the concept regarding the biological optic system, which allows the customized treatment of the refractive errors in order to obtain predictable and reliable refractive outcomes. This desired goal has been made possible by the technological innovations in ophthalmologic surgery, which have opened new perspectives difficult to imagine in the past. There are numerous choices, which can be considered by the surgeon to correct even the most difficult refractive errors, namely: intraocular lenses with personalized design, phakic intraocular lenses, femtosecond LASER refractive surgery, or BIOPTICS.

BIOPTICS is an association of surgical techniques approaching the two main refractive planes of the anterior pole (cornea and lens) for the correction of unusual refractive errors and postsurgical refractive “surprises”.

The BIOPTICS principle is based on both a holistic vision of refraction and on the refractive uniqueness of each patient. It combines the phakic of pseudophakic intraocular lens implantation with a corneal LASER refractive procedure, therefore represents a double intervention (which also named this procedure). Practically, the sequence of interventions could be previously planned or adopted as a necessity for the fine refractive adjustment.

In 1994, Dr. Zaldivar first introduced the concept, consisting of a LASIK procedure after a phakic intraocular implant for high myopia. Also in 1994, Dr. Guell defined for the first time the term “adjustable refractive surgery” through LASIK to correct residual ametropia after various ocular surgeries: phakic implant, pseudophakic implant or perforant keratoplasty. Presently, this is the meaning of BIOPTICS technique: IOL implant (phakic or pseudophakic – monofocal, multifocal, accommodative), followed by a wavefront guided corneal refractive LASER (LASIK, i-LASIK, PRK), for refractive errors that cannot be corrected only by one procedure, for example: high hyperopia, high myopia, greater than 5D astigmatism or to correct residual refractive errors (after pseudophakia implant or after VISIAN ICL).

It is obvious than in cases of extreme refractive errors, total correction in a single refractive plane is insufficient. Therefore, dividing the procedure into two planes of optical correction has the following advantages: maintaining a wider optical area, implantation of thinner IOLs, a better predictability of the target refraction, lower subjective complication with better functional results. A special indication of the BIOPTICS method is the phakic implant for high hyperopia with accommodative effects difficult to preoperatively estimate and a lower accuracy of the refractive outcome that needs adjustment through a subsequent corneal refractive procedure. There is another aspect: patients with very high refractive errors demand early spectacle independence and a superior visual quality. At young ages, they usually choose a first corrective intervention consisting in phakic implant and the subsequent evolution of the refractive error imposes a supplementary refractive surgery.

Lens surgery (opacified through cataract or clear lens extraction for refractive purpose) is another chapter in which the BIOPTICS technique has an important role. With the disappearance of the boundary between cataract surgery and refractive surgery, IOL implant became synonym with a perfect vision for patients.

Preoperative calculation formulas for refraction are mathematical formulas that only approximate (more and more exactly, but not perfectly) the physical optics. In reality, sometimes, the complexity of ocular refraction overcomes the biometric investigation methods and the technology of premium implants. Moreover, the human and biological factor still surprise through unexpected refractive outcomes.

Postoperative astigmatism could be caused by postoperative variation of the implant position, manufacturing implant defects, intraocular toric implant rotation or ignoring the back corneal astigmatism. Various anatomical conditions, for example myopic scleral staphyloma or the intraocular presence of silicon oil could lead to such biometric inaccuracy. In these cases of refractive “surprises”, the residual unwanted diopters are eliminated through customized corneal LASER ablation in order to obtain emmetropia.

Patients with implants for presbyopia correction are extremely sensitive to residual refractive errors (especially to astigmatism that is lower even to 1D). The vision quality deterioration is strongly perceived and this perception is subjectively accentuated by the greater postoperative visual expectation of these patients. They start having reading and nighttime vision difficulties and thus the functional outcome is unsatisfactory. At this moment, the surgeon could use BIOPTICS to eliminate the unwanted diopter.

Lens and corneal surgery are reciprocally sustained through diminishing the secondary effects and complications related to each particular procedure: selection of optical parameters, that create an optical area with a safe dimension at each level, reduces glare, halos, or optical and chromatic aberrations. There is also the risk of summing certain complications, which affect the result of these two procedures. For each patient, the preoperative evaluation should lead to an optimal selection of technique combination in order to get at the end emmetropia without functional and anatomical complication.

VISIAN ICL implant (Implantable Collamer Lens) is another high-quality method for extreme refractive errors correction. This is a pliable spherical or toric intraocular implant, which places the phakic eye through a 3.5mm corneal incision in the posterior chamber of the eye. It is made of a polymeric biocompatible material named collamer, which is capable of absorbing the ultraviolet light. Its design protects the lens anterior capsule through an anterior arch. The optic area has a convex-concave shape and the haptics are flat to facilitate sub-iris insertion in the posterior chamber. The optical area functions as a refractive plane in order to reduce the refractive error (myopia, hyperopia, astigmatism); the implant could be spherical or toric.

The contraindications are anterior chamber depth under 3mm, iridocorneal angle lower than 30 degrees, low density of endothelial cells and patient age under 21 years old. In case of high myopia, the refractive outcome is precise for values up to -15D. When the diopter exceeds -18D, the correction separation between ICL implant and cornel ablation through BIOPTICS, is a solution that should be taken into account. The diameter of the optical part varies with the implant diopter power and the correction distribution between the implant and cornea allows the selection of parameters convenient from the point of view of postoperative side effects such as halos or unsatisfactory night vision.

In conclusion, at present there is a wide range of sophisticated procedures to correct refractive errors. Necessity? The need for perfection? Refinement? Undoubtedly, a little bit of everything could be found in each technique, in order to offer a perfect vision adapted to the patient’s needs, especially to the ones with high refractive errors. In many cases, the actual optical status of the patient imposes a wisely selection of these procedures, because the ocular biology could offer challenges and “surprises” to which both the surgeon and the patient have to deal with.

**Prof. Dr. Tataru Calin Petru M.D.,Ph.D.**

